# Epidemiology of medical emergency team calls in patients undergoing lung transplantation

**DOI:** 10.1016/j.jhlto.2026.100605

**Published:** 2026-06-04

**Authors:** Judit Orosz, Vinodh Bhagyalakshmi Nanjayya, Greg Snell, Bronwyn Levvey, Helen Shingles, Samantha Ennis, David Pilcher, Daryl Jones

**Affiliations:** a Alfred Intensive Care Academic Centre, Bayside Health, Prahran, Victoria 3004, Australia; b Lung Transplant Service, Alfred Hospital Bayside Health, Prahran, Victoria 3004, Australia; c Central Clinical School, Monash University, Melbourne, Australia; d The Australian and New Zealand Intensive Care Research Centre, School of Public Health and Preventive Medicine, Monash University, St. Kilda Rd, Prahran, Victoria 3004, Australia; e Department of Intensive Care, Austin Hospital, Austin Health, Heidelberg, Victoria, Australia

**Keywords:** Lung transplantation, ICU readmission, Medical Emergency Team, Transplantation characteristics, Organ support

## Abstract

**Background:**

Patients undergoing lung transplantation (LTx) are at risk of clinical deterioration during hospital ward care. The epidemiology of Medical Emergency Team (MET) calls in LTx patients and their association with patient outcomes are poorly described.

**Purpose:**

We aimed to (1) measure the frequency, timing, and characteristics of MET activations in patients undergoing LTx, (2) evaluate differences in demographics and outcomes between those who had a MET call vs those who didn’t.

**Methods:**

A retrospective cohort study of patients who received MET calls during their index admission for LTx at the Alfred Hospital between January 2016 and December 2021.

**Results:**

Amongst 535 patients undergoing LTx, MET activation occurred in 265 (49.5%), with 738 MET activations in total. The MET group had a median (IQR) of 2 (1-4) MET calls. Pre-LTx period accounted for 15% of the calls, with hypoxia being the most common trigger (49.5%). During the post-LTx period, tachycardia (28.5%) and hypotension (33.1%) were the most common triggers. Hospital length-of stay (LOS) was longer in the MET group (26 [19-41] vs 19 days [15-27], *p* < 0.001), and ICU readmission occurred more frequently (27.5 vs 2.2%, *p* < 0.001). Post LTx MET activation had no impact on 90-day mortality [Adj HR (95% CI): 1.41 (0.19-10.60); *p* = 0.74] or on long-term survival [Adj HR (95% CI): 1.09 (0.80-1.49); *p* = 0.59].

**Conclusions:**

MET activation is common in patients undergoing LTx. Admissions with MET are associated with higher rates of ICU readmission and longer hospital LOS compared to the no MET group, without affecting survival outcomes.

## Background

Lung transplantation (LTx) is performed in end-stage lung disease to improve survival with quality of life.^1^ Pulmonary fibrosis, chronic obstructive pulmonary disease, and pulmonary vascular disease are the most common indications for LTx.^2^, ^3^ In Australia, LTx numbers have increased in the last decade with a concurrent improvement in survival. In bilateral LTx recipients, survival at 1, 3, and 5 years are 90%, 74%, and 68%, respectively.^4^

In the setting of increasingly older recipients with high co-morbidity burden, patients undergoing LTx are often medically unwell^5^, ^6^ and at risk of clinical deterioration during their hospital stay. This complex group requires an Intensive Care Unit (ICU) stay post-LTx and patients frequently stay on the ward for a prolonged period, requiring significant resources after ICU discharge.^7^

Medical Emergency Teams (METs) are interdisciplinary teams typically led by the ICU who identify and treat deteriorating patients on the hospital wards.^8^ METs or similar structures are now implemented worldwide after the Joint Commission described the need to improve recognition and response to changes in a patient’s condition in its patient safety goals in 2008^9^ and are mandatory in Australia as part of hospital accreditation.^10^ The MET can be activated by anyone based on predefined criteria.

The etiology and timing of clinical deterioration on the ward in LTx patients has not been well described. Previous studies described the characteristics of ICU readmissions from the ward after LTx.^11^, ^12^ It is understood that in-hospital mortality of general inpatients is increased if patients develop abnormal clinical observations or have a MET activation.^13^, ^14^, ^15^ Abnormal vital signs are common before in-hospital cardiac arrests on hospital wards.^16^ On the other hand, in a recent review, survival rates were similar amongst liver transplant patients reviewed by the MET compared with those who did not require MET review, although the median length of hospital stay was longer in patients who experienced MET activation.^17^

We aimed to characterize the epidemiology of MET calls in LTx patients in a high-volume LTx center with a well-established MET system. The objectives of this study were to (1) measure the frequency of MET activations in patients undergoing LTx, (2) describe the timing and characteristics of MET calls in relation to the timing of LTx, and (3) describe the differences in demographics and outcomes between those who had a MET call vs those who did not.

## Methods

### Study design

We conducted a retrospective cohort study of MET calls that occurred before and after LTx in patients during the index admission (patient population) in the Alfred Hospital between January 2016 and December 2021.

### Setting

The Alfred Hospital is a tertiary teaching Hospital in Melbourne, Victoria, Australia.

It is one of the 4 adult LTx services in Australia and the nationwide pediatric LTx referral service, run conjointly with the Royal Children’s Hospital, Melbourne.^2^ The Victorian Lung Transplant Service accepts referrals primarily from the states of Victoria, Tasmania, and South Australia (total population 9.5 million). Following LTx, all patients are admitted to the ICU. After ICU discharge, patients are managed by dedicated LTx physicians on the respiratory ward, with ICU follow-up initially and ongoing multidisciplinary input from multiple teams.

The hospital has a well-established MET system, which was introduced in December 2000.^18^ The MET reviews patients on the ward, and activation is mandatory once patients meet predefined criteria (Appendix Table A1). The calling criteria can only be modified with the approval of the treating team. The attending team consists of an ICU liaison nurse and the primary team (lung Tx) registrar in hours, as well as an ICU registrar after hours, and as needed during the day. The MET is not activated while the patient stays in the ICU, and overall, the MET attends approximately 8,000 calls a year hospital-wide. While MET calls occur frequently in the organization, the cardiac arrest (Code Blue) rate (where there is loss of cardiac output with initiation of chest compressions and /or defibrillation) is low.^19^ (In the pediatric age group, all calls are called Code Blue.)

### Ethics

The study was reviewed by the Alfred Hospital Ethics and Research Committee, and low-risk ethics approval was obtained with a waiver of informed consent (Approval number 327/22).

### Participants

All patients who underwent LTx during the study period were included. Patients who were admitted for the purpose of LTx but never received a transplant were not included in the study.

### Data sources

Patients were identified from the prospectively collected local LTx Clinical Database. The database contains information on donor and recipient characteristics. Data related to all hospital admission characteristics, including ICD diagnosis codes were extracted from the electronic medical records (Cerner Millennium/ Oracle, Kansas, USA). Data routinely collected for submission to the Australian and New Zealand Intensive Care Society (ANZICS) clinical quality registry was extracted from the local ICU database.^20^ This contained basic demographics, chronic comorbidities, clinical frailty score (CFS),^21^ illness severity score (APACHE III), interventions provided in the ICU, and outcomes. Data related to all MET calls and characteristics was obtained from the Victorian Health Incident Management System (VHIMS), a standardized dataset, where all MET and Code Blue (cardiac arrest) call characteristics are documented.

### Statistical analysis

For continuous variables, mean with standard deviation (SD) or median with interquartile range (IQR) were used to report the summary statistics, depending on the distribution. Categorical data was summarized as number with proportion (%). Unpaired *t*-test or Wilcoxon rank sum tests were used to compare the continuous variables based on the distribution. The categorical data were compared using Fisher’s exact test. In addition, survival analysis using the Kaplan-Meier estimator and Cox proportional hazards model was performed to assess the effect of MET calls and other covariates on both short-term (90-day) and long-term survival following LTx. Patients were censored on the day of data extraction from the lung transplant database.

Hazard ratios (HR) with 95% confidence intervals (CI) were calculated to assess the impact of covariates on both short-term and long-term survival. The proportional hazards assumption was verified using Schoenfeld residuals. Kaplan-Meier survival curves were plotted to visualize differences between the groups with the MET calls vs no MET calls, and log-rank tests were used to compare survival distributions. The covariates included in the analysis were age, sex, Charlson Comorbidity Index, BMI (Body Mass Index), 6-minute walk test prior to transplantation, waitlist days, epidural analgesia, whether the transplant was done on bypass, ICU length of stay, APACHE III-J score (Acute Physiology and Chronic Health Evaluation), organ supports needed in ICU (ECMO, NIV, renal replacement therapy), and afterhours discharge. All tests were conducted at a 2-sided alpha level of 0.05. All statistical analysis was performed using Stata 18.0 SE (StataCorp, College Station, Texas).

## Results

**Figure 1:**
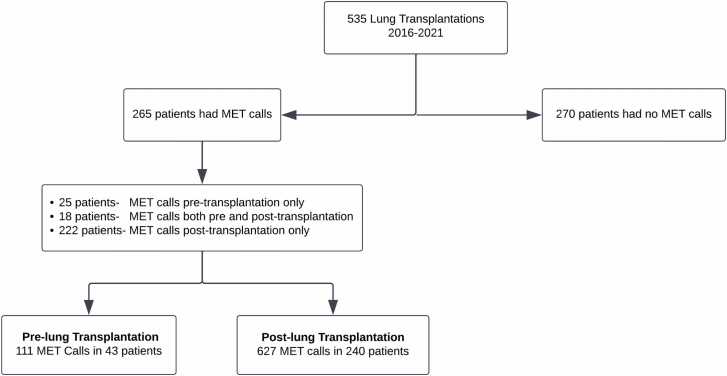
Flow diagram of lung transplant recipients by Medical Emergency Team activation status between 2016 and 2021. MET, Medical Emergency Team.

During the 6-year study period, 535 patients underwent LTx, of whom 265 (49.5%) had 738 MET activations during the index admission with a median rate of (IQR) 2^1^, ^2^, ^3^, ^4^ MET calls (Figure 1).

### Patient demographics and organ support (Table 1)

The mean (SD) age was 54.3 (15.64) years, and 59.6% of the patients were male. Table 1 summarizes the demographic and admission characteristics of patients by MET call status. The comorbidity burden was moderate, as assessed by the Charlson comorbidity index^22^ in both groups. A history of cardiac arrhythmias occurred more often as a comorbidity amongst patients with MET calls. Median 6-minute walk test distance was lower amongst patients who had MET call(s) (295.5 vs 328 m, *p* = 0.02). 78.2% of the patients had at least mild frailty, as measured by a Clinical Frailty Score (CFS) ≥5 at the time of hospital admission, although there was no significant difference in CFS between the MET call vs No MET call groups.

**Table 1:** Demographic and Admission Characteristics by Medical Emergency Team Activation Status

Variable	Overall (*N* = 535)	MET call (*N* = 265)	No MET call (*N* = 270)	*p*-value
Age (years), mean (SD)	54.3 (15.64)	55.3 (14.91)	53.4 (16.31)	0.17
Age < 18 years, n *(%)*	23 (4.3)	10 (3.8)	13 (4.8)	0.67
Male, *n* (%)	319 (59.6)	159 (60.0)	160 (59.3)	0.86
BMI (kg/m^2^), mean (SD)	23.8 (4.2)	23.6 (4.1)	23.9 (4.3)	0.45
6MWT (m), median (IQR)*	309 (240-385)	295.5 (232-363)	328 (240-400)	0.02
Mild or more severe frailty (CFS ≥5), *n* (%)	329 (78.2)	166 (77.3)	163 (79.6)	0.64
*Co-morbidities, n (%)*				
Heart Failure	65 (13.0)	36 (14.8)	29 (11.2)	0.29
Cardiac Arrhythmias	219 (43.7)	145 (59.7)	74 (28.7)	<0.001
Pulmonary Circulation Disease	109 (21.8)	54 (22.2)	55 (21.3)	0.83
Hypertension	72 (14.4)	40 (16.5)	32 (12.4)	0.20
Diabetes	288 (53.8)	144 (54.3)	144 (53.3)	0.86
*Co-morbidities Index Scores, median (IQR)*				
Updated Charlson Comorbidity	1 (1-2)	1 (1-2)	1(1-2)	0.09
*ICU Admission Characteristics*				
APACHE III Score, mean (SD)	56.7 (16.53)	56.4 (15.99)	57.1 (17.07)	0.63
Duration of Mechanical ventilation (hours), median (IQR)	37 (19-89)	39 (20-87)	33(18-89)	0.27
Tracheostomy, *n* (%)	40 (7.5)	20 (7.6)	20 (7.4)	1.00
NIV, *n* (%)	131 (24.5)	67 (25.3)	64 (23.7)	0.69
ECMO, *n* (%)	62 (11.6)	31 (11.7)	31 (11.5)	1.00
Renal Replacement therapy, *n* (%)	52 (9.7)	26 (9.8)	26 (9.6)	1.00
ICU LOS (days), median (IQR)	4.8 (3-8.9)	4.8 (3.1-9.2)	4.7 (2.9-8.7)	0.19
After-hours discharge from ICU, *n* (%)	151 (28.2)	74 (27.9)	77 (28.5)	0.92

*Data missing for six-minute walk test in 44 patients-(25 in MET call and 19 in No MET call group).

Co-morbidities data missing in 44 patients-22 in MET call and 12 in No MET call, APACHE III data missing in 8 patients-1 in MET call and 7 in no MET call, Frailty data missing in 115 patients-50 in MET call and 65 no MET call group.

6MWT, 6-minute walk test; APACHE, Acute Physiology and Chronic Health Evaluation; BMI, Body Mass Index; CFS, Clinical Frailty Score; ECMO, extracorporeal oxygenation; ICU, Intensive Care Unit; IQR, interquartile range; LOS, length of stay; MET, Medical Emergency Team; NIV, noninvasive ventilation; SD, standard deviation.

The intensity of ICU therapy was also similar between the 2 groups regarding the frequency of non-invasive ventilation (NIV), continuous renal replacement therapy, and ECMO. There was no significant difference between the ICU length of stay.

### Transplantation characteristics and outcomes (Table 2)

The indications for transplantation were similar in the MET call group and in the No MET call group, with obstructive lung disease and restrictive lung disease being the most common indications in both groups. The transplant surgery characteristics were similar between the 2 groups. Numerically, higher proportion of patients in the MET call group returned to the theater compared to the No MET call group, but this difference did not reach statistical significance (30.6% vs 23.7%, *p* = 0.08).

**Table 2:** Lung Transplantation Characteristics and Outcomes by Medical Emergency Team Activation Status

Variable	Overall (*N* = 535)	MET call (*N* = 265)	No MET call (*N* = 270)	*p*-value
Days on waiting list, median (IQR)	70 (27-174)	69 (27-191)	70 (28-161)	0.77
ECMO as a bridge to transplantation, *n* (%)	15 (2.8)	10 (3.8)	5 (1.9)	0.20
*Indication for lung transplantation, n (%)*				
Obstructive lung disease	189 (35.3)	101 (38.1)	88 (32.6)	0.28
Restrictive lung disease	183 (34.2)	94 (35.5)	89 (33)	
Septic lung disease	69 (12.9)	32 (12.1)	37 (13.7)	
Pulmonary Hypertension	46 (8.6)	20 (7.5)	26 (9.6)	
Re-transplantation	48 (9.0)	18 (6.8)	30 (11.1)	
*Lung Transplantation Characteristics, n (%)*				
Single lung	74 (13.8)	36 (13.6)	38 (14.1)	0.90
Double lung	447 (83.6)	223 (84.2)	224 (83.0)	
Combined*	14 (2.6)	6 (2.3)	8 (3.0)	
Donation after Cardiac Death Donor, *n* (%)	152 (28.4)	73 (27.6)	79 (29.3)	0.70
Transplant done on cardiac bypass, *n* (%)	155 (29.0)	82 (30.9)	73 (27.0)	0.34
Cardiac bypass duration (minutes), median (IQR)	169 (124-218)	169 (135-223)	168 (117-208)	0.35
Thoracic Epidural analgesia, *n* (%)	396 (74.0)	195 (73.6)	201 (74.4)	0.84
Return to theater, *n* (%)	145 (27.1)	81 (30.6)	64 (23.7)	0.08
*Outcomes*				
ICU Readmission, *n* (%)	79 (14.8)	73 (27.5)	6 (2.2)	<0.001
Hospital LOS, (days) median (IQR)	22 (17-35)	26 (19-41)	19 (15-27)	<0.001
Mortality at 3 months, *n* (%) **	21 (3.9)	7 (2.6)	14 (5.2)	0.18
Mortality at 12 months, *n* (%)	43 (8.0)	23 (8.7)	20 (7.4)	0.64
*Discharge destination, n (%)*				
Home/Private residence	443 (94.2)	209 (91.7)	234 (96.7)	0.005
Rehabilitation	23 (4.9)	18 (7.9)	5 (2.1)	
Residential aged care facility	4 (0.9)	1 (0.4)	3(1.2)	

*Combined group includes 7 (1.3%) Lung-kidney, 5 (0.95%) heart-lung, and 1 (0.19%) lung-liver transplantations, 1 (0.19%) Lung-CABG combined surgery.

** The 7 deaths which occurred in the MET call group, occurred in ICU-4 of them occurred during the first ICU admission and 3 deaths occurred during the second ICU admission following MET calls.

ECMO, Extracorporeal membrane oxygenation; IQR, Interquartile range; MET, Medical emergency team.

ICU readmission rate was significantly higher in the MET call group during the admission (27.5% vs 2.2%, *p* < 0.001). The hospital length of stay was significantly longer in the MET call group compared to the no MET call group [median (IQR) 26 (19-41) days in MET call group vs 19 (15-27) days in no MET call group]. All deaths occurred in the ICU in the study population, and there was no statistically significant difference in the 3-month (2.6% vs 5.2%; *p* = 0.18) or 12-month mortality (8.7% vs 7.4%, *p* = 0.64) between the 2 groups.

### Medical emergency team activation characteristics

The characteristics of MET activations are summarized in Table 3. Overall, 738 MET calls were called for 265 of the 535 patients—an activation rate of 1,379 MET calls per 1,000 LTx admissions. There were 7 cardio-respiratory arrests in total during the study period. One in 4 patients (26.1%) had multiple MET calls during the index admission. Relatively few patients (18 patients) had MET calls in both the pre- and post-LTx period.

**Table 3:** Characteristics of the Medical Emergency Team Calls

Variables	Overall (*N* = 738)	Pre-lung Transplantation (*N* = 111)	Post-lung Transplantation (*N* = 627)	*p*-value
Transplant to MET call, duration (days), median (IQR)*	9.9 (5.8-23.3)	13.5 (4.8-27)	12.1 (7.6-26.9)	0.32
After-hours MET call, *n* (%)	260 (35.2)	43 (38.7)	217 (34.6)	0.45
Weekend MET calls, *n* (%)	215 (29.1)	30 (27.0)	185 (29.5)	0.65
Cardio-respiratory arrest, *n* (%)	7 (0.9)	0 (0.0)	7 (1.1)	0.60
Multiple MET calls, *n* (%)	192 (26.1)	22 (19.8)	170 (27.2)	0.13
Reason for MET Call, n (%) *				
Tachycardia, HR>140/min	183 (24.9)	5 (4.5)	178 (28.5)	<0.001
SBP <90 mmHg	219 (29.8)	12 (10.8)	207 (33.1)	<0.001
SBP >200 mmHg	1 (0.1)	0 (0.0)	1 (0.2)	1.00
Respiratory Failure - RR>36	109 (14.8)	24 (21.6)	85 (13.6)	0.04
Respiratory Failure - SpO2<90% on O2	170 (23.1)	55 (49.5)	115 (18.4)	<0.001
GCS drop or recurrent/prolonged seizures	36 (4.9)	7 (6.3)	29 (4.6)	0.47
Pediatric	8 (1.1)	1 (0.9)	7 (1.1)	1.00
Serious Concern	75 (10.2)	15 (13.5)	60 (9.6)	0.23
Uncontrolled Bleeding	5 (0.7)	2 (1.8)	3 (0.5)	0.17
Uncontrolled Pain	27 (3.7)	2 (1.8)	25 (4.0)	0.41
*Goals of care at the time of MET call, n (%)*				
For full resuscitation with standard MET criteria	334 (48.4)	29 (27.1)	305 (52.3)	<0.001
For full resuscitation with modified MET criteria	130 (18.8)	28 (26.2)	102 (17.5)	
Limited resuscitation and standard MET criteria	12 (1.7)	10 (9.3)	2 (0.3)	
For limited resuscitation with modified MET call criteria	21 (3)	18 (16.8)	3 (0.5)	
Unknown	183 (28)	22 (20.6)	171 (29.3)	
*Disposition After MET call, n (%)*				
Stayed on ward for active management	617 (83.8)	92 (82.9)	525 (84.0)	<0.001
Transferred to higher acuity ward	32 (4.3)	1 (0.9)	31 (5.0)	
Readmitted to ICU	75 (10.2)	15 (13.5)	60 (9.6)	
Transferred to OR	5 (0.7)	3 (2.7)	2 (0.3)	
Transferred to ED	3 (0.4)	0 (0.0)	3 (0.5)	
Paperwork not completed	3 (0.4)	0 (0.0)	3 (0.5)	

*Multiple reasons can occur at the same MET.

ED, Emergency Department; GCS, Glasgow Coma Scale; HR, heat rate; ICU, Intensive Care Unit; IQR, Interquartile range; MET, Medical Emergency Team; OR, Operating Room; RR, respiratory rate; SBP, systolic blood pressure.

111 (15 % of 738) MET calls occurred in the pre-LTx period in 43 patients. Prior to transplantation, two thirds of MET activations were for abnormal respiratory observations (a fall in oxygen saturation in 49.5 %, and tachypnoea in 21.6% of the pre-transplantation activations). Limited resuscitation goals (pre-agreed life-sustaining interventions that the patient can receive) and modified MET criteria were more common in the pre- transplantation period. The majority of patients stayed on the ward after MET activation both pre- and post-transplant and did not require ICU admission.

**Figure A1:**
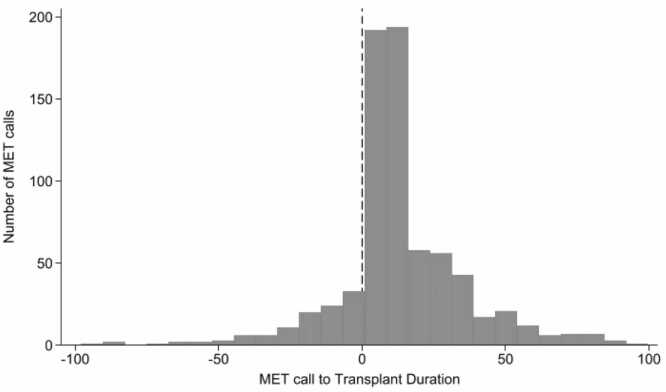
Distribution of the MET calls in relation to the lung transplant surgery.

Most MET calls (627 calls or 85%) occurred in the post-transplantation period. The patterns of MET calls were different in the post-transplantation period, with the most common triggers for MET in the post-transplantation period being hypotension (33.1%) and tachycardia (28.5%). Even though respiratory triggers for METs were less common than in the pre-LTx period, they still occurred commonly post-LTx. Tachypnoea or desaturation listed as at least one of the reasons in 1 in 3 post-LTx METs altogether. Post-LTx, modifications in resuscitation status and MET calling criteria were less common. MET calls for other than abnormal vital signs were less common (serious concern of clinical deterioration, uncontrolled bleeding, pain, etc.). The median time to MET call post-LTx was 12.1 days. The distribution of MET calls in relation to the timing of the LTx is shown on (Appendix Figure A1).

**Figure A2:**
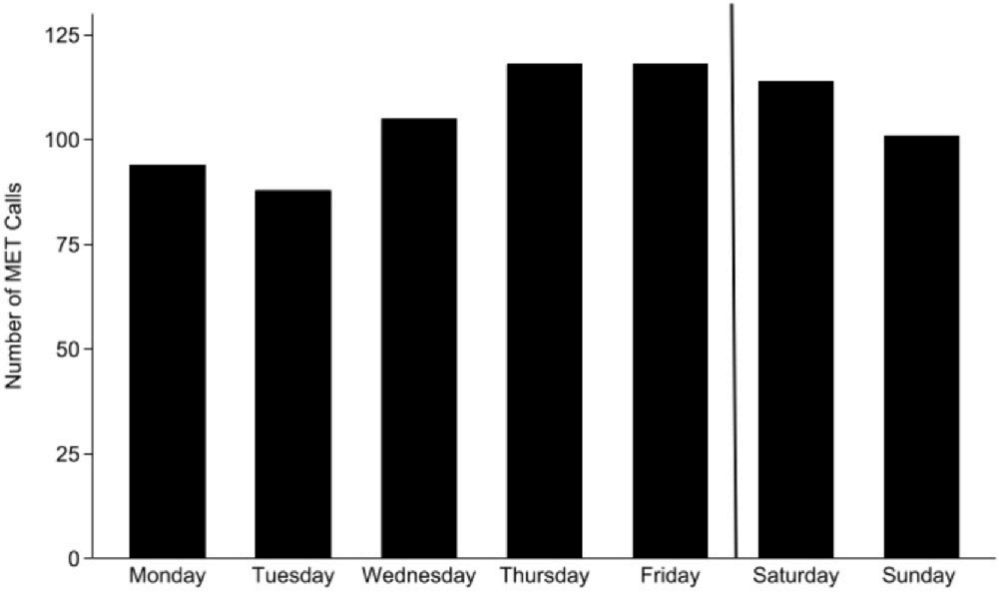
Distribution of MET calls over the day of the week.

There was a trend of increased MET calls during the weekend (Appendix Figure A2).

### Association of postoperative MET calls with 90-day survival

We assessed the relationship of the post LTx MET calls with 90-day survival *(*Table 4). On univariate Cox regression analysis, the main factors associated with higher hazard of death within 90-days were higher Charlson comorbidity index score, perioperative ECMO support, and renal replacement therapy. On multivariate Cox regression analysis, Charlson comorbidity index and perioperative ECMO support were the main risk factors associated with lower 90-day survival. There was no statistically significant difference between the MET call and no MET call groups in univariate and multivariate Cox regression analysis (Adjusted HR (95% CI): 1.41 (0.19-10.60); *p* = 0.74).

**Table 4:** Factors Associated With 90-Day Survival Following Lung Transplantation

Variable	Univariate analysis		Multivariate analysis	
	HR (95% CI)	*p*-value	HR (95% CI)	*p*-value
Age, years	1.00 (0.95-1.06)	0.95	-	
Gender				
Female	Ref			
Male	1.01(0.17-6.07)	0.98	-	
Updated Charlson Comorbidity Index	1.73 (1.14-2.62)	0.01	1.61 (0.93-2.76)	0.09
6MWT, m	0.99 (0.98-1.00)	0.13	-	
Waitlist days	1.00 (0.99-1.00)	0.25	-	
Epidural analgesia				
No	Ref			
Yes	0.48 (0.08-2.84)	0.42	-	
Cardiopulmonary bypass				
No	Ref			
Yes	0.65 (0.07-5.85)	0.70		
APACHE III score	0.99 (0.94-1.05)	0.82	-	
ECMO				
No	Ref		Ref	
Yes	8.59 (1.21-60.99)	0.03	8.25 (1.14-9.51)	0.04
Renal Replacement Therapy				
No	Ref			
Yes	11.14 (1.57-79.07)	0.02	-	
ICU length of stay, days	1.28 (0.52-3.16)	0.60	-	
After-hours discharge				
No	Ref			
Yes	0.64 (0.07-5.72)	0.69	-	
Hospital LOS, days	1.77 (0.48-6.52)	0.39	-	
MET call				
No	Ref		Ref	
Yes	1.75 (0.29-10.47)	0.54	1.41 (0.19-10.60)	0.74

6MWT, six-minute walking test; APACHE, Acute Physiology and Chronic Health Evaluation; BMI, Body mass index; CI, Confidence interval; ECMO, Extracorporeal membrane oxygenation; HR, Hazard ratio; MET, Medical Emergency Team; Ref, reference category.

### Sensitivity analysis

**Figure 2:**
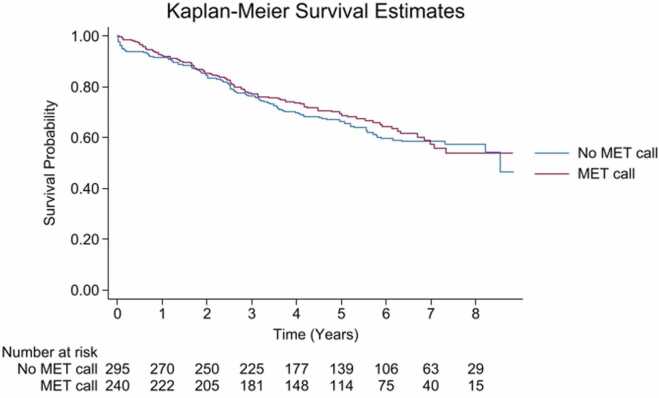
Long term survival between the MET calls vs No MET calls groups*. Adjusted Hazard Ratio (95% CI): 1.09 (0.80-1.49); *p* value = 0.59. *For this analysis, patients who had MET calls only in the post-lung transplantation period are included in the MET call group. Those who had MET calls only during the pre-lung transplantation period (*n* = 25) are included in the no MET call group (based on their postoperative MET call status). MET, Medical Emergency Team; CI, Confidence interval.

We also performed a sensitivity analysis to assess the association between postoperative MET calls and the long-term survival in patients after LTx (Appendix Table A2). Postoperative MET call status had no impact on long-term survival after hospital discharge up to the censor day (Figure 2). Multivariate Cox regression analysis identified sex, type of LTx, and APACHE III score as the main factors associated with long-term survival after hospital discharge.

## Discussion

In this single-center retrospective cohort study evaluating the epidemiology of MET calls during the LTx admissions, we found that MET activation occurred in 1 in 2 patients with an overall rate of 1,379 MET calls per 1,000 LTx admissions. Most of the MET calls occurred during the post-transplantation period. Readmission rate to the ICU was higher in patients with MET calls, and they had a longer hospital length of stay compared to patients who did not need MET activations. Patients with postoperative MET call activation had similar survival outcomes compared to patients with no postoperative MET calls.

Rapid Response Teams and METs have been shown to reduce in-hospital mortality and cardiac arrest rates outside ICU.^23^, ^24^, ^25^ Higher Rapid Response Teams utilization rates (number of calls per 1,000 admissions) result in higher reduction in mortality, suggesting a dose-response effect, as previously demonstrated.^26^ We report a rate of 1379 MET calls per 1000 lung transplant admissions, which is one of the highest MET rates reported in the literature. In comparison, the frequency of MET activations in all patients who were discharged alive from ICU was 19% in the organization in 2021, with an ICU readmission rate of 6%.

The high rate of MET activations can be explained by the single-parameter activation system and mandatory calls in our organization once patients reach pre-determined vital sign abnormalities. One of the challenges of the single parameter activation system is the high number of alerts. An Australian retrospective single center study examining the incidence of meeting triggers for multi-tiered escalation response during the entire hospital stay found that 41.3% of the patients triggered at the highest level of escalation in the Medical Emergency Tier.^27^ This is consistent with our findings. Most MET calls in our study were triggered by abnormal vital signs, indicating that abnormal vital signs occurred very frequently in this patient group during their ward stay.

ICU readmission was common in the MET group. In our hospital, MET activation has become a major pathway to refer patients to ICU and to obtain timely ICU input. Kim et al examined the readmission rate in LTx patients in a single-center study and found that 24.5% of patients got readmitted to the ICU which is higher than the overall readmission rate in our study (14.8%) despite the high rate of MET calls in our hospital.^11^ We presume the single parameter MET system with high activation rate and consistent response reliably identified the deteriorating patients in this patient population. Post LTx the median time to MET was 12.1 days. With a median ICU length of stay of 4.86 days, this suggests calls happened well after the index ICU discharge. We presume, therefore, that new physiological abnormality caused the MET activation in most of the cases, rather than abnormal physiology persisted after the ICU or patients were discharged too early from the ICU.

The median hospital length of stay was longer in the MET call group. A single-center study examining MET calls on liver transplant patients also found that the median length of stay was longer in patients who experienced MET activation.^17^

The most common trigger for MET activation in the post-LTx period was tachycardia and hypotension. While we cannot comment on the specific type of tachyarrhythmias in our cohort, arrhythmias are well described in this patient population. A single-center study on atrial arrhythmias early after transplantation reported that 25% of the patients developed atrial arrhythmias within 30 days in the post-transplant.^28^, ^29^

When we examined the effect of post LTx MET calls on short-term mortality, we found that the risk of 90-day mortality was not significantly increased in the MET call group. This is consistent with the fact that most deaths occurred during the index ICU admission and not after ICU discharge. Those who survived to ICU discharge were likely to survive to hospital discharge.

Our study provides evidence that early recognition and management of deterioration on the ward may improve patient outcomes. The overall 12-month survival was 92% in the study cohort, which exceeds internationally reported 12-month survival rates of approximately 89%.^30^ We use a patient-centered multidisciplinary model of care in all aspects of recipient management, from assessment and waitlisting to pre-, peri- and post-operative care.^31^ Patients need to meet discharge criteria before transfer to the respiratory ward. The ICU team remains involved in the management of patients, providing post discharge follow-up ward rounds and reviews during MET calls or as requested. Our findings suggest that high use of the MET as an integral part of patient management for LTx patients in our hospital may minimize failure to rescue and may improve patient outcomes. During the study period there were no unexpected deaths on the ward following LTx.

### Strengths and limitations

Our study describes accurately recorded MET activation rates in a large lung transplant center in Australia with a well-established MET system. Identifying common triggers for MET activation can help identifying common problems that occur in the post-transplantation period and could inform guideline development to address common causes of deterioration.

The study has several limitations, including a single-center study design and retrospective data collection. Local differences in culture around MET activation and early ICU readmission may limit generalizability. Furthermore, we have no information on the pathophysiology that caused the abnormal vital signs (e.g., sepsis, pain etc.), or how many patients were managed in monitored areas other than the ICU for a period. Additional intra- and postoperative factors and other unaccounted confounders could also affect postoperative trajectories. Further research is needed to assess the causes of abnormal vital signs and the optimal and most efficient management of abnormal vital signs in patients undergoing LTx. The results of the study provide information to determine staffing and monitoring requirements on wards managing LTx patients.

## Conclusions

MET activation is common during hospital admission for LTx, particularly in the post-LTx period. Patients with MET have higher ICU readmission rates and longer hospital length of stay compared to patients with No MET. However, MET call patients had similar short and long-term survival.

## Conflicts of Interest statement

The authors declare that they have no known competing financial interests or personal relationships that could have appeared to influence the work reported in this paper.

## Figures and Tables

**Table A1:** MET Call/ Code Blue Criteria – Adult (>18 Years)

**Vital sign**	**MET call**	**Code Blue**
Airway	Threatened	Not breathing or absence of normal breathing
Respiratory rate	≤ 6 breaths/min **OR** ≥ 36 breaths/min	
Oxygen saturation (on air or on O2)	< 90% (on air or oxygen)	
Oxygen flow rate	Oxygen flow rate ≥ 8 l/min (excludes HFNP) FIO_2_ ≥ 0.5 on Venturi mask/NIV or HFNP	
Systolic blood pressure	< 90 mmHg **OR** ≥ 200 mmHg	
Heart rate	≤ 40 beats/min **OR** ≥ 140 beats/min	
Conscious state	Any unexpected decrease in level of consciousness Fall in GCS > 2 points Seizures	Unresponsive Absence of movement
Other	Serious concern Uncontrolled pain	

**Table A2:** Factors associated with long term survival following lung transplantation*

**Variable**	**Univariate Analysis**		**Multivariate Analysis**	
	**HR (95% CI)**	***p*-value**	**HR (95% CI)**	***p*-value**
Age	1.01 (0.995-1.02)	0.32	-	
Gender				
Female	Ref		Ref	
Male	0.80 (0.59-1.07)	0.14	0.72 (0.53-0.97)	0.03
BMI (kg/m^2^)	1.02 (0.98-1.05)	0.33	-	
6MWT (m)	1.00 (1.00-1.00)	0.09	-	
Waitlist days	1.00 (1.00-1.00)	0.40	-	
Transplantation type				
Single lung	Ref		Ref	
Double lung	0.50 (0.34-0.71)	<0.01	0.44 (0.30-0.64)	<0.001
Combined	0.13 (0.02-0.96)	0.046	0.10 (0.01-0.76)	0.03
Cardiopulmonary Bypass				
No	Ref		-	
Yes	1.14 (0.83-1.59)	0.41	-	
APACHE 3 score	1.02 (1.01-1.03)	<0.001	1.02 (1.01-1.03)	<0.001
Inotropes				
No	Ref			
Yes	0.69 (0.25-1.88)	0.47	-	
ECMO				
No	Ref			
Yes	0.86 (0.50-1.49)	0.60	-	
Renal replacement therapy				
No	Ref			
Yes	0.89 (0.48-1.64)	0.71	-	
After-hours discharge				
No	Ref			
Yes	1.36 (1.00-1.86)	0.05	1.33 (0.97-1.83)	0.08
ICU LOS (days)**	1.12 (0.94-1.32)	0.20	-	
ICU Readmission				
No	Ref			
Yes	1.30 (0.87-1.93)	0.20	-	
Delirium				
No	Ref			
Yes	1.17 (0.74-1.84)	0.51	-	
Pressure Injury				
No	Ref			
Yes	0.82 (0.33-2.01)	0.67	-	
MET call				
No	Ref			
Yes	1.06 (0.79-1.42)	0.70	1.09 (0.80-1.49)	0.59
Hospital LOS	1.37 (1.07-1.15)	0.03	1.23 (0.94-1.61)	0.14

*Date of censoring is the date of analysis 27/11/24. The analysis only includes patients who were alive at the time of hospital discharge.

**ICU LOS and Hospital LOS are log transformed in the analysis.

6MWT, six-minute walking test; APACHE, Acute Physiology and Chronic Health Evaluation; BMI, Body mass index; CI, Confidence interval; ECMO, Extracorporeal membrane oxygenation; HR, Hazard ratio; MET, Medical Emergency Team; Ref, reference category.

## References

[1] Bos S., Vos R., Van Raemdonck D.E., Verleden G.M. (2020). Survival in adult lung transplantation: Where are we in 2020?. Curr Opin Organ Transpl.

[2] Chambers D.C., Perch M., Zuckermann A. (2021). The International Thoracic Organ Transplant Registry of the International Society for Heart and Lung Transplantation: thirty-eighth adult lung transplantation report - 2021; Focus on recipient characteristics. J Heart Lung Transpl.

[3] Christie J.D., Van Raemdonck D., Fisher A.J. (2024). Lung transplantation. N Engl J Med.

[4] Paraskeva M.A., Levin K.C., Westall G.P., Snell G.I. (2018). Lung transplantation in Australia, 1986-2018: more than 30 years in the making. Med J Aust.

[5] Varughese R., Rozenberg D., Singer L.G. (2020). An update on frailty in lung transplantation. Curr Opin Organ Transpl.

[6] Shigemura N., Toyoda Y. (2020). Elderly patients with multiple comorbidities: insights from the bedside to the bench and programmatic directions for this new challenge in lung transplantation. Transpl Int.

[7] Banga A., Mohanka M., Mullins J. (2017). Hospital length of stay after lung transplantation: independent predictors and association with early and late survival. J Heart Lung Transpl.

[8] Jones D.A., DeVita M.A., Bellomo R. (2011). Rapid-response teams. N Engl J Med.

[9] (2007). The Joint Commission announces the 2008 National Patient Safety Goals and Requirements. Jt Comm Perspect.

[10] (2021). Australian Commission on Safety and Quality in Health Care.

[11] Kim H.B., Na S., Paik H.C., Joo H., Kim J. (2021). Risk factors for intensive care unit readmission after lung transplantation: a retrospective cohort study. Acute Crit Care.

[12] Cohen J., Singer P., Raviv Y. (2011). Outcome of lung transplant recipients requiring readmission to the intensive care unit. J Heart Lung Transpl.

[13] Buist M., Bernard S., Nguyen T.V., Moore G., Anderson J. (2004). Association between clinically abnormal observations and subsequent in-hospital mortality: a prospective study. Resuscitation.

[14] Goldhill D.R., McNarry A.F. (2004). Physiological abnormalities in early warning scores are related to mortality in adult inpatients. Br J Anaesth.

[15] Psirides A.J., Hill J., Jones D. (2016). Rapid response team activation in New Zealand hospitals-a multicentre prospective observational study. Anaesth Intensive Care.

[16] Andersen L.W., Kim W.Y., Chase M. (2016). The prevalence and significance of abnormal vital signs prior to in-hospital cardiac arrest. Resuscitation.

[17] Robertson M., Lim A.K.H., Bloom A. (2021). Epidemiology and prognostic significance of rapid response system activation in patients undergoing liver transplantation. J Clin Med.

[18] Jones D.A., Mitra B., Barbetti J., Choate K., Leong T., Bellomo R. (2006). Increasing the use of an existing medical emergency team in a teaching hospital. Anaesth Intensive Care.

[19] Bingham G., Bilgrami I., Sandford M. (2018). Avoiding adult in-hospital cardiac arrest: a retrospective cohort study to determine preventability. Aust Crit Care.

[20] Stow P.J., Hart G.K., Higlett T. (2006). Development and implementation of a high-quality clinical database: the Australian and New Zealand Intensive Care Society Adult Patient Database. J Crit Care.

[21] Subramaniam A., Ueno R., Tiruvoipati R., Srikanth V., Bailey M., Pilcher D. (2022). Comparison of the predictive ability of clinical frailty scale and hospital frailty risk score to determine long-term survival in critically ill patients: a multicentre retrospective cohort study. Crit Care.

[22] Charlson M.E., Pompei P., Ales K.L., MacKenzie C.R. (1987). A new method of classifying prognostic comorbidity in longitudinal studies: development and validation. J Chronic Dis.

[23] Winters B.D., Weaver S.J., Pfoh E.R., Yang T., Pham J.C., Dy S.M. (2013). Rapid-response systems as a patient safety strategy: a systematic review. Ann Intern Med.

[24] Maharaj R., Raffaele I., Wendon J. (2015). Rapid response systems: a systematic review and meta-analysis. Crit Care.

[25] Solomon R.S., Corwin G.S., Barclay D.C., Quddusi S.F., Dannenberg M.D. (2016). Effectiveness of rapid response teams on rates of in-hospital cardiopulmonary arrest and mortality: a systematic review and meta-analysis. J Hosp Med.

[26] Jones D., Bellomo R., DeVita M.A. (2009). Effectiveness of the medical emergency team: the importance of dose. Crit Care.

[27] Flabouris A., Nandal S., Vater L., Flabouris K., O'Connell A., Thompson C. (2015). Multi-tiered observation and response charts: prevalence and incidence of triggers, modifications and calls, to acutely deteriorating adult patients. PLoS One.

[28] Orrego C.M., Cordero-Reyes A.M., Estep J.D. (2014). Atrial arrhythmias after lung transplant: underlying mechanisms, risk factors, and prognosis. J Heart Lung Transpl.

[29] Chaikriangkrai K., Jyothula S., Jhun H.Y. (2015). Incidence, risk factors, prognosis, and electrophysiological mechanisms of atrial arrhythmias after lung transplantation. JACC Clin Electrophysiol.

[30] Gottlieb J., Sybrecht C., Holm A.M. (2025). Heart-lung transplantation-global activity between 2003 and 2023, indications and outcomes. JHLT Open.

[31] Paraskeva M.A., Westall G.P., Pilcher D. (2014). The Alfred Hospital Lung Transplant Experience. Clin Transpl.

